# The Investigation and Management of Peri-Prosthetic Joint Infection After Total Knee Arthroplasty: An Update Based on the Latest British Orthopaedic Association Standard and Speciality Standard Guidelines

**DOI:** 10.7759/cureus.73315

**Published:** 2024-11-09

**Authors:** Sizar Doski, Alexandra Sebastiao, Prashant Thayaparan

**Affiliations:** 1 Emergency Medicine, Imperial College Healthcare NHS Trust, London, GBR; 2 Trauma and Orthopaedics, Royal Free London NHS Foundation Trust, London, GBR; 3 Trauma and Orthopaedics, Whittington Health NHS Trust, London, GBR

**Keywords:** british orthopaedic association, british orthopaedic association standards, peri-prosthetic joint infection, primary knee replacement, tka (total knee arthoplasty), total knee replacement complications

## Abstract

Peri-prosthetic joint infection (PJI) is a significant complication following total knee arthroplasty (TKA). Early identification and management are essential to prevent severe morbidity and mortality in these patients. Long-term complications of PJI include the need for multiple operations, disability, joint stiffness, reduced range of motion, and increased mortality. Clinical signs, inflammatory markers, imaging, tissue sampling, and synovial fluid analysis are required to diagnose PJI. Debridement antibiotics and implant retention (DAIR) is an effective management option, but single- or two-stage exchange arthroplasty may be ultimately required. All cases of PJI in TKA must be discussed in a multi-disciplinary (MDT) meeting. This review incorporates the updated British Orthopaedic Association (BOA) standard and speciality standard to provide an up-to-date guideline on the early identification and management of PJI. We highlight that adhering to the BOA guidelines and adopting an MDT approach are essential for optimal patient outcomes.

## Introduction and background

Peri-prosthetic joint infection (PJI) is a significant complication in patients who undergo total knee arthroplasty (TKA) [1]. The National Joint Registry (NJR) recorded 226,350 total knee replacements between 2018 and 2020 in England, Wales, Northern Ireland, the Isle of Man, and the States of Guernsey, and these numbers are on the rise [2,3]. Therefore, prompt identification and management of this condition is essential to prevent severe morbidity and mortality, as well as preserve prosthetic function [4,5]. The British Orthopaedic Association (BOA) Standard: Acute Management of Peri-Prosthetic Joint Infection was released in 2023 to identify life-threatening sepsis and immediate recognition of PJI [6]. This was followed by the BOA Specialty Standards (SpecS) Peri-Prosthetic Joint Infection for Definitive Management in 2024 [7], underlying the importance of early identification and management of PJI. Our literature review primarily aims to present up-to-date information on epidemiology, clinical features, investigations, and management options for PJI in TKA based on the recent BOA guidelines. The secondary aim is to consolidate our findings into a flowchart, which, we believe, will assist clinicians in identifying and managing PJI as per the BOA guidelines. 

Epidemiology

The incidence of PJI after TKA ranges from 0.5% to 1.8% in primary procedures [8,9]. The risk of developing a PJI is highest in the first two years postoperatively [3,9,10]. In the NJR, recorded between April 2003 and December 2020, 23.5% of TKR revisions were performed due to an infection (n=20,527), second only to aseptic loosening/lysis (33.6%, n=29,387) [3,11]. The NJR’s prosthesis time incident rates (PTIR) estimates for revision due to infection show 0.94 (95% CI: 0.92-0.96) revisions per 1,000 prosthesis years for all cases of TKA, second only to aseptic loosening. This number is 1.95 (1.87-2.02) per 1,000 prosthesis years due to infection in the first year, 0.48 (0.45-0.52) in years five to seven, and 0.28 in years 13-15 post-primary procedure [3].

## Review

Aetiology

The presence of a foreign body significantly reduces the concentration of bacteria required to cause an infection and increases the possibility of biofilm formation. The organism is protected from host defences in this state with the extracellular component, composed of polysaccharides and proteins [1,10]. The most commonly isolated organisms in PJI are Staphylococcus aureus and coagulase-negative staphylococci [12], seen in around 50-60% of PJI. Staphylococcus aureus is most likely to present early in the postoperative period in primary arthroplasty, defined as a PJI within three months postoperatively [13]. The study by Tai et al. shows that a positive culture for enterococcus, corynebacterium species, fungi, or mycobacteria may suggest a polymicrobial infection [13].

Pathogens can infect prosthetic joints through three different identified mechanisms:

Direct Inoculation

This accounts for the majority of PJI that occurs within the first postoperative year. This occurs during the prosthetic joint implantation phase of surgery, through contamination of the prosthesis or peri-prosthetic tissue from direct contact or air-borne contamination [1].

Contiguous Spread

This occurs when pathogens spread to the prosthetic joint from adjacent infected tissue. Incomplete healing of the superficial and deep fascial planes postoperatively can lead to surgical site infections (SSI), which may subsequently lead to PJI [1,10]. Contiguous spread can cause delayed PJI secondary to trauma or surgery in the surrounding tissue area [1].

Haematogenous Spread

This occurs when pathogens spread via the blood from other body sites to the prosthetic joint. Although rare, there is an estimated risk of 30-40% of haematogenous spread with staphylococcus aureus bacteraemia [14]. Late infections (after 12 months) generally arise from haematogenous spread [1,12]. Bacteraemia due to less virulent organisms such as enterococci and coagulase-negative staphylococci usually presents from 3-12 months [15]. Streptococci, associated with delayed and late presentation, and enterococci, are associated with 10% of PJI infections combined [10].

Risk factors 

Risk factors associated with PJI include malnutrition [16], immunosuppression, and underlying rheumatological conditions [1]. Diabetes mellitus, hyperglycaemia, alcohol use, and malignancy are also implicated in PJI, as is revision surgery following the primary implantation [10]. Perioperative infections of distant sites, such as urinary or pulmonary infections, are associated with PJI in TKA [10]. Infection risk is around 1.8 times after primary knee TKA in both current and former smokers [17].

Long-term complications

PJI significantly impacts patient health and quality of life due to long-term complications. PJI can lead to suboptimal outcomes, multiple operations, long periods of disability, and an increased risk of mortality. Mortality is reportedly higher in the elderly or if the isolated species is either enterococci or methicillin-resistant Staphylococcus aureus (MRSA) [18].

As a result of repeated surgeries or prolonged infection, patients may present with joint stiffness and reduced range of motion, which severely limits daily activities and mobility [19]. Treatment of prosthetic joint infection requires long-term antibiotics, debridement and retention of implants, or revisional surgery performed in one-stage or two-stage [12]. This process is expensive and prolonged, and its impact on patients and their families is substantial [2]. 

Clinical features

Acute PJI presentations vary depending on the mode of infection, the affected joint and the soft tissue around the joint [10]. Acute PJI after TKA is characterised by the onset of arthralgia, swelling, erythema, and warmth. Systemic signs, including a fever, may be present. Wound drainage, or a sinus tract, may accompany it [19,20]. Chronic PJI after TKA may present with more subtle clinical features. Patients often report discomfort, persistent arthralgia, stiffness, and joint instability. Sinus tract formation can also be associated with chronic infection [10]. 

Earlier classification systems defined infections within three months of arthroplasty as early, those presenting between 3 and 24 months as delayed, and those after two years as late [20]. The current suggested classifications are based partly on timing but incorporate mode of infection, haematogenous spread, bone and soft tissue defects responsible pathogen, and the systemic host status - which may correspond to factors such as age, neutropenia or other risk factors [20,21]. 

Investigation and examination 

A thorough history and examination are essential in patients presenting with suspected PJI following TKA, with interval observations of vital signs. Prosthesis surgery date, implant brand and size, operation notes, and any postoperative complications should be documented. All subsequent infections should be reported, including recent antibiotic use. The orthopaedic surgeon must investigate other sources of infection, including endocarditis [6]. Clinicians should not start antibiotics until an orthopaedic surgeon is consulted unless the patient is haemodynamically unstable from sepsis [7]. 

Initial investigations in acute PJI without evidence of sepsis include C-reactive protein (CRP), full blood count (FBC), renal function, and plain radiographs of the affected joint [6]. PJI should not be ruled out with normal inflammatory markers if clinical suspicion remains high, especially in the presence of immunosuppression [6]. An orthopaedic consultant should assess the patient within 48 hours in a stable or systemically well patient. Empirical antibiotics should only be commenced after peri-prosthetic tissue and fluid sampling [6,7,22].

**Figure 1:**
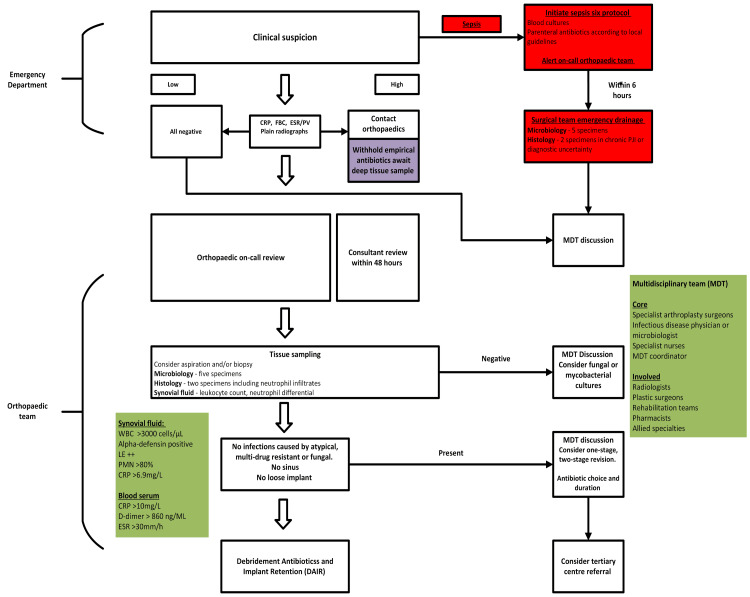
Suggested treatment algorithm for managing PJI in TKA Initial management follows the British Orthopaedic Association (BOA) Acute Management of PJI guidelines, in the emergency department. Immediate recognition and resuscitation are essential. The BOA speciality standards highlight the importance of the orthopaedic speciality roles. The synovial fluid and blood serum markers highlight the definition agreed upon by the International Consensus Meeting CRP: C-reactive protein; ESR: erythrocyte sedimentation rate; FBC: full blood count; LE: leukocyte esterase; MDT: multi-disciplinary team; PJI: peri-prosthetic joint infection; PMN: polymorphonuclear; PV: plasma viscosity; TKA: total knee arthroplasty; WBC: white blood cells Image Credit: Sizar Doski

In the presence of sepsis, BOA Standards for Trauma and Orthopaedics (BOAST) guidelines highlight the importance of initiating the "Sepsis Six" protocol immediately and alerting the on-call orthopaedic team. The sepsis protocol [23] must include parenteral antibiotics, according to local guidelines, and patients should have blood cultures taken. The orthopaedic clinician must aspirate the affected joint, preferably within six hours [6]. If debridement is indicated, five microbiological and two histological samples should be taken. A suggested management plan in the emergency department is summarised in Figure 1 based on the BOAST acute PJI guideline from 2023 [6].

Following the initial management, the orthopaedic team must ensure multidisciplinary input throughout the treatment process; these include consultant radiologists, microbiologists, physiotherapists and district nurses. Mandatory investigations include FBC, renal function, CRP, and serological analysis, including erythrocyte sedimentation rate (ESR) or plasma viscosity (PV), with plain radiographs. A CT and MRI may help with evaluation and discussion in MDT [26].

Plain radiographs may show displacement of the prosthesis, the components, and osteolysis. However, it can't differentiate between septic and aseptic osteolysis [1]. CT scans may demonstrate infection with fluid collection or periosteal reaction, with good diagnostic performance [26]. MRI is particularly accurate in detecting purulent infection and peri-prosthetic osteolysis [1,26]. The SpecS guidelines do not recommend a bone scan routinely; however, it suggests that newer nuclear imaging techniques may be helpful in diagnostic uncertainty [7]. Tissue sampling should be undertaken with five separate microbiological and two histological samples. A synovial fluid leucocyte count, neutrophil differential, or additional markers such as leucocyte esterase should also be conducted [7]. The suggested definitive management technique is summarised in Figure 1. 

Parvizi et al. have proposed a scoring-based definition for PJI, an update of the International Consensus Meeting (ICM) for the definition and diagnosis of PJI in 2013, which was formalised in ICM 2018 [24,25,27]. This incorporates the major criteria for PJI diagnosis defined by the Musculoskeletal Infection Society (MSIS). Major criteria for diagnosis of PJI include one of the following: either a sinus tract formation communicating with the joint or two cultures with the same isolated pathogen. In the minor and additional criteria, the scoring system incorporates blood serum, synovial, and histological analysis to aid the diagnosis of PJI [24]. The BOA speciality standard for investigating and managing PJI in TKA recommends diagnosis using standardised criteria of the ICM 2013 [22,25].

Differential diagnosis 

Differentiating aseptic complications of a TKA is the primary challenge encountered when diagnosing PJI. Common symptoms in the immediate postoperative phase such as erythema, swelling, pain, and joint stiffness can resemble PJI [28]. Aseptic cases of TKA dysfunction may also include osteolysis, increased wear or debonding of cement. However, it is essential to rule out PJI since approximately 12% of aseptic joint dysfunctions have an underlying PJI [29,30]. Continuous pain is associated with PJI, whereas pain on mobilising or weight bearing is associated with aseptic failure [1].

Synovial fluid analysis may help differentiate between hemarthrosis or other crystal arthropathies, such as gout and calcium pyrophosphate deposition [28]. Spinal radiculopathy, vascular claudication, tendinopathy or local bursitis, and systemic conditions, such as autoimmune disease, may result in joint pain that resembles PJI [28].

Management

Debridement antibiotics and implant retention (DAIR) can help effectively manage PJI and may treat around 60% of cases [5]. However, DAIR is contraindicated in the presence of a sinus, atypical, fungal, or multidrug-resistant organisms. The infected joint must not show signs of loosening. If the patient is immunocompromised, the performing surgeon must take caution with DAIR as it may not be indicated [7]. Single- or two-stage exchange arthroplasty is necessary following failed management with DAIR. This is a decision that should be taken by an MDT. Arthroscopic washout and debridement should only be used in an emergency, i.e., if mortality is an immediate risk; they have no place in the definitive management of PJI [7,22]. 

Complex cases involving previous revision for a PJI, multi-drug resistant bacteria, or fungal infections should be discussed in the MDT and referred to a tertiary centre [22]. The referring team may use the Revision Knee Complexity Classification (RKCC) system to evaluate complex cases to determine if a referral is indicated [31].

## Conclusions

Prompt identification and management of acute PJI is critical to preserve joint function and prevent morbidity and mortality. It should be considered in any patient who presents with a painful, hot, swollen knee with or without drainage after primary or revision TKA. Key clinical features include acute pain, erythema, and discharge; patients may also show systemic signs of illness. Prompt identification and immediate resuscitation are essential in the presence of sepsis. Blood inflammatory markers and plain radiographs are necessary for initial investigations. This should be followed by a comprehensive assessment by the orthopaedic team and deep tissue sampling. DAIR is an effective definitive management if indicated, whilst other surgical interventions may include single-stage or two-stage revision surgery. Of note, acute PJI management should always involve a coordinated MDT.
